# Spinous Process Osteochondroma as a Rare Cause of Lumbar Pain

**DOI:** 10.1155/2016/2683797

**Published:** 2016-08-04

**Authors:** Bárbara Rosa, Pedro Campos, André Barros, Samir Karmali, Esperança Ussene, Carlos Durão, João Alves da Silva, Nuno Coutinho

**Affiliations:** ^1^Trauma and Orthopaedics Department, Hospital Vila Franca de Xira, 2600 009 Lisbon, Portugal; ^2^Department of Pathology, Hospital Vila Franca de Xira, 2600 009 Lisbon, Portugal

## Abstract

We present a case of a 5th Lumbar Vertebra (L5) spinous process osteochondroma as a rare cause of lumbar pain in an old patient. A 70-year-old male presented with progressive and disabling lower lumbar pain. Tenderness over the central and left paraspinal area of the lower lumbar region and a palpable mass were evident. CT scan showed a mass arising from the spinous process of L5. Marginal resection of the tumor was performed through a posterior approach. The histological study revealed an osteochondroma. After surgery, pain was completely relieved. After one year there was no evidence of local recurrence or symptoms. Osteochondromas rarely involve the spine, but when they do symptoms like pain, radiculopathy/myelopathy, or cosmetic deformity may occur. The imagiologic exam of election for diagnosis is CT scan. When symptomatic the treatment of choice is surgical resection. The most concerning complication of osteochondromas is malignant transformation, a rare event.

## 1. Introduction

Osteochondroma is a benign outgrowth of bone and cartilage and is one of the most common bone tumors that usually occurs in long bones but rarely involves the spine [1], affecting mainly the cervical and upper dorsal segments [2]. They are more common in males and have an average age at presentation of approximately 32 ± 4.6 years [3]. Lumbar osteochondromas can be asymptomatic or cause symptoms like pain, radiculopathy/myelopathy, or cosmetic deformity [3–10]. The imagiologic exam of election for diagnosis is CT scan [4, 11]. When symptomatic the treatment of choice is surgical resection. The most concerning complication of osteochondromas is malignant transformation, a rare event [2, 12].

We have found in the literature one case of a symptomatic lumbar osteochondroma presenting in the 6th decade of life [5]. We report a case of a lumbar osteochondroma presenting in the 8th decade of life causing lumbar back pain. Despite being rare, we must consider osteochondroma as a cause of lumbar back pain, even in older patients.

## 2. Case Report

**Figure 1:**
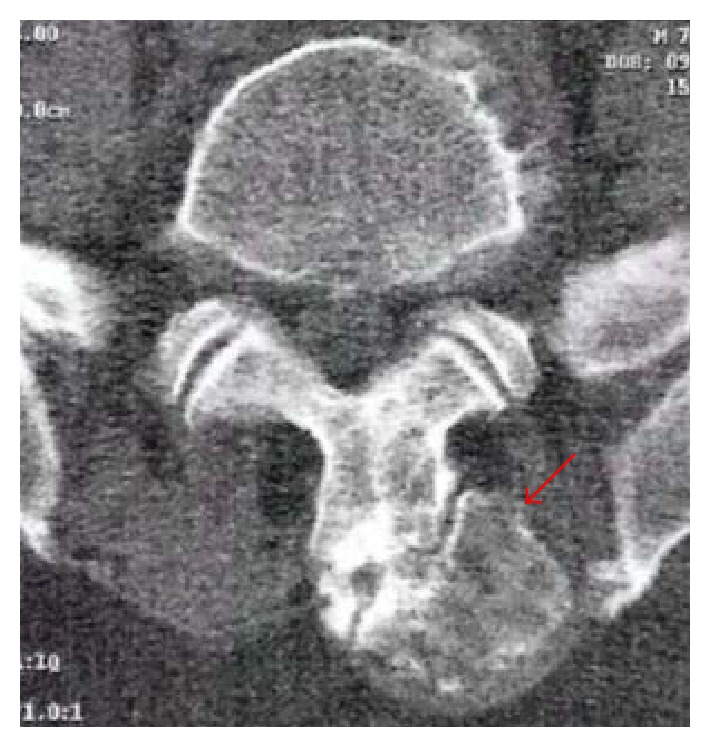
CT scan. Axial view showing a well-limited mass with a cartilage cap arising from the spinous process of L5 lateralized to the left (arrow).

**Figure 2:**
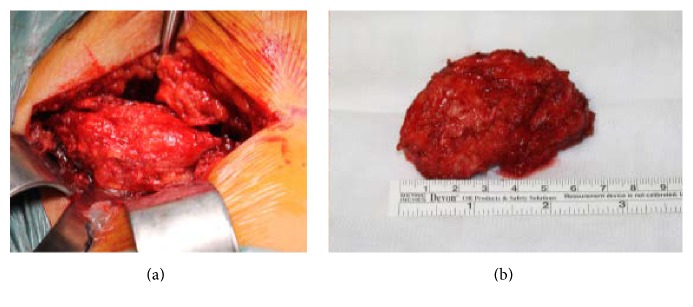
(a) Intraoperative picture showing a lumbar spine midline approach, exposing the tumor* in situ* in contiguous to the spinous process of L5 causing adjacent left paraspinal muscular compression. (b) Intraoperative picture of the resected tumor with an approximately 7 cm axis-length.

**Figure 3:**
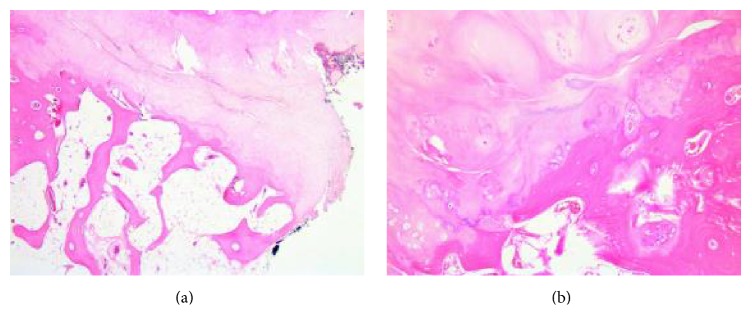
(a) Hematoxylin-eosin stain, original magnification ×2.5, and (b) Hematoxylin-eosin stain, original magnification ×10. Junction of cartilage cap and underlying bone without atypia and resemblance to an epiphyseal plate with enchondral ossification.

A 70-year-old male, with history of hypertension, dislipidemia, and hyperuricemia, presented to our institution with a one-year long history of progressive and intense lower lumbar pain causing great limitation of daily activities. Physiotherapy or medication was ineffective. The patient reported a palpable mass on this region for years but with neither symptoms nor size progression. He had no constitutional or neurologic symptoms. On examination, there were tenderness over the central and left paraspinal area and a fixed palpable mass of size approximately 7 × 5 cm, hard in consistency, and no pulse. The pain aggravated with flexion, extension, and rotational trunk movements. Neurologic examination was normal. Radiographs showed a bony mass protruding posteriorly, apparently from the L5 vertebra. CT scan showed a 7 cm long well-limited mass with an apparent cartilage cap arising from the spinous process of L5. It was lateralized to the left with adjacent paraspinal muscle compression (Figure 1). Under general anesthesia, the tumor was marginally resected along with the L5 spinous process through a posterior approach (Figure 2). Histologic examination has shown a specimen composed of trabecular bone with focus on bone marrow covered by lobules of cartilaginous tissue, without cellular atypia, consistent with osteochondroma (Figure 3). After surgery pain was completely relieved, and neurologic function was normal. At one-year follow-up there was no evidence of local recurrence or symptoms.

## 3. Discussion

Osteochondroma is a benign outgrowth of bone and cartilage and is one of the most common bone tumors that usually occurs in long bones but rarely involves the spine [1]. Only 1,3% to 4,1% of solitary osteochondromas arise in spine and occur in approximately 9% of patients who are affected by hereditary multiple exostosis [4, 11]. They are more common in males and according to Gaetani et al. [3] the average age at presentation is approximately 32 ± 4.6 years. Only five cases of lumbar osteochondroma out of 17 occurred in the L5 level. We have found in the literature one case of a symptomatic lumbar osteochondroma presenting in the 6th decade of life [5]. Until now, the oldest case of spinal osteochondroma reported in the literature occurred in a 73-year-old female in the cervical spine [2]. We report a case of a lumbar osteochondroma presenting in the 8th decade of life.

The tumors are thought to arise through a process of progressive endochondral ossification of aberrant cartilage of a growth plate following surgery or fracture or as a consequence of a congenital perichondrial deficiency and are the most common radiation-induced benign tumors [1, 4, 6]. Choi et al. [5] reported a case in which the osteochondroma arose from a spondylolytic lamina and speculate that the fibrous cartilage of spondylolysis served as the origin of aberrant cartilaginous tissue.

The tumor affects mainly the cervical and dorsal spine, probably related to different durations of the ossification processes that occur in the secondary centers of ossification. It can be speculated that the more rapidly the ossification process of these centers develops, the greater the probability that aberrant cartilage will form is. In adolescence, secondary ossification centers, which lie in the spinous process, transverse process, articular process, and the endplate of vertebral body, complete the growth of the vertebral column. These secondary ossification centers appear in children between the ages of 11 and 18 years. They develop into complete ossification in the cervical spine during adolescence and in the thoracic and the lumbar spine during the end of the second decade of life [4, 13].

In most reported cases we have found in the current literature, involving the lumbar spine, the tumor is included in posterior arch elements, more commonly the lamina [3–10, 14]. We have found only two reported cases like this one with involvement of the spinous process [7, 14].

The tumor can be asymptomatic or symptomatic, either causing pain by pressure on adjacent soft tissue structures when it grows posteriorly, or, more rarely, causing radicular or spinal compression symptoms, when it grows into the spinal canal [3, 4, 6–9]. The tumor can also cause cosmetic deformity, as occurred in a case of an 8-year-old girl presenting with an atypical spinal curvature caused by a lumbar osteochondroma [10].

Marrow and cortical continuity with the underlying parent bone defines the lesion [6] and this feature is better visualized on computed tomography scan [4]. MRI is useful to determine the extent of neurologic structures compromise and it identifies lesions that look suspicious of malignant transformation [6].

When symptomatic, the treatment of choice of osteochondromas is surgical resection. However, Gille et al. [2] recommend systematic surgical resection of all solitary spinal osteochondromas, given the risk of malignant transformation. The resection can be achieved in the majority of cases without spinal instrumentation because it rarely compromises the spinal stability, as osteochondromas show focal growth in the posterior elements. We have found only one case reported on which fusion and instrumentation surgery was necessary [5].

The most concerning complication of osteochondromas is malignant transformation, fortunately a rare complication. Chondrosarcoma of the spine represents 4–10% of all chondrosarcomas and 12% of all malignant tumors of the spine [15]; the frequency of degeneration is estimated at about 1% in solitary spinal osteochondromas [16]. Altay et al. [12] in a retrospective analysis of 627 cartilage-forming tumors revealed a rate of malignant transformation for solitary osteochondromas of 4,2% and a higher rate for multiple osteochondromas, namely, 9,2%. However, none of these tumors involve the spine. Malignant transformation leads to a chondrosarcoma in 90% of cases, which develops in the cartilage cap of the osteochondroma. The most consistent finding that may suggest malignancy might be a cap thickness >2 cm, but the diagnosis is only confirmed with a biopsy of the lesion [12, 17].

## 4. Conclusion

We report a case of a lumbar osteochondroma arising from the L5 spinous process, a rare cause of lumbar pain, especially in the 8th decade. Osteochondromas rarely involve the spine, but when they occur they can be asymptomatic or cause symptoms, like pain, radiculopathy or myelopathy, or, even, cosmetic deformation. The imagiologic exam of election for diagnosis is CT scan. When symptomatic the treatment of choice is surgical resection. The most concerning complication of osteochondromas is malignant transformation, fortunately a rare event.
